# Brucellosis: an overview.

**DOI:** 10.3201/eid0302.970219

**Published:** 1997

**Authors:** M J Corbel

**Affiliations:** National Institute of Biological Standards and Control, South Mimmas, Potters Bar, Hertfordshire, United Kingdom. mcorbel@nibsc.ac.uk

## Abstract

Brucellosis remains a major zoonosis worldwide. Although many countries have eradicated Brucella abortus from cattle, in some areas Brucella melitensis has emerged as a cause of infection in this species as well as in sheep and goats. Despite vaccination campaigns with the Rev 1 strain, B. melitensis remains the principal cause of human brucellosis. Brucella suis is also emerging as an agent of infection in cattle, thus extending its opportunities to infect humans. The recent isolation of distinctive strains of Brucella from marine mammals has extended its ecologic range. Molecular genetic studies have demonstrated phylogenetic affiliation to Agrobacterium, Phyllobacterium, Ochrobactrum, and Rhizobium. Polymerase chain reaction and gene probe development may provide more effective typing methods. Pathogenicity is related to production of lipopolysaccharides containing a poly N-formyl perosamine O chain, CuZn superoxide dismutase, erythrlose phosphate dehydrogenase, stress-induced proteins related to intracellular survival, and adenine and guanine monophosphate inhibitors of phagocyte functions. Protective immunity is conferred by antibody to lipopolysaccharide and T-cell-mediated macrophage activation triggered by protein antigens. Diagnosis still centers on isolation of the organism and serologic test results, especially enzyme immunoassay, which is replacing other methods. Polymerase chain reaction is also under evaluation. Therapy is based on tetracyclines with or without rifampicin, aminoglycosides, or quinolones. No satisfactory vaccines against human brucellosis are available, although attenuated purE mutants appear promising.


### Brucellosis: an Overview

Brucellosis remains a major zoonosis worldwide. Although many countries have eradicated *Brucella abortus* from cattle, in some areas *Brucella melitensis* has emerged as a cause of infection in this species as well as in sheep and goats. Despite vaccination campaigns with the Rev 1 strain, *B. melitensis* remains the principal cause of human brucellosis. *Brucella suis* is also emerging as an agent of infection in cattle, thus extending its opportunities to infect humans. The recent isolation of distinctive strains of *Brucella* from marine mammals has extended its ecologic range. Molecular genetic studies have demonstrated the phylogenetic affiliation to *Agrobacterium, Phyllobacterium, Ochrobactrum,* and *Rhizobium.* Polymerase chain reaction and gene probe development may provide more effective typing methods. Pathogenicity is related to production of lipopolysaccharides containing a poly N-formyl perosamine O chain, Cu-Zn superoxide dismutase, erythrulose phosphate dehydrogenase, stress-induced proteins related to intracellular survival, and adenine and guanine monophosphate inhibitors of phagocyte functions. Protective immunity is conferred by antibody to lipopolysaccharide and T-cell-mediated macrophage activation triggered by protein antigens. Diagnosis still centers on isolation of the organism and serologic test results, especially enzyme immunoassay, which is replacing other methods. Polymerase chain reaction is also under evaluation. Therapy is based on tetracyclines with or without rifampicin, aminoglycosides, or quinolones. No satisfactory vaccines against human brucellosis are available, although attenuated *purE* mutants appear promising.

Brucellosis has been an emerging disease since the discovery of *Brucella melitensis* by Bruce in 1887. Subsequently, an increasingly complex pattern of strains has emerged with the identification of *Brucella abortus, Brucella suis, Brucella neotomae, Brucella ovis, Brucella canis*, and, more recently, types infecting marine mammals. Because each type has distinctive epidemiologic features, with each new type, the complexity of the interaction with humans has increased. Because new strains may emerge and existing types adapt to changing social and agricultural practices, the picture remains incomplete.

This synopsis reviews major advances in the knowledge of certain aspects—genetics, antigenic structure, mechanisms of pathogenicity, diagnosis, treatment, and prevention of the disease—of the *Brucella* genus and its host interactions.

### Epidemiology

Worldwide, brucellosis remains a major source of disease in humans and domesticated animals. Although reported incidence and prevalence of the disease vary widely from country to country, bovine brucellosis caused mainly by *B. abortus* is still the most widespread form (Tables 1-5). In humans, ovine/caprine brucellosis caused by *B. melitensis* is by far the most important clinically apparent disease. The disease has a limited geographic distribution, but remains a major problem in the Mediterranean region, wes-tern Asia, and parts of Africa and Latin America. Recent reemergence in Malta and Oman indicates the difficulty of eradicating this infection (1). Sheep and goats and their products remain the main source of infection, but *B. melitensis* in cattle has emerged as an important problem in some southern European countries, Israel, Kuwait, and Saudi Arabia. *B. melitensis* infection is particularly problematic because *B. abortus* vaccines do not protect effectively against *B. melitensis* infection; the *B. melitensis* Rev.1. vaccine has not been fully evaluated for use in cattle. Thus, bovine *B. melitensis* infection is emerging as an increasingly serious public health problem in some countries. A related problem has been noted in some South American countries, particularly Brazil and Colombia, where *B. suis* biovar 1 has become established in cattle (2). In some areas, cattle are now more important than pigs as a source of human infection.

Address for correspondence: National Institute of Biological Standards and Control, Blanche Lane, South Mimms, Potters Bar, Hertfordshire EN6 3QG, United Kingdom; fax: 44-1-707-646-730; e-mail: mcorbel@nibsc.ac.uk.

Table 1. Brucellosis in animals, Europe, 1994

| Country | Bovine (*B. abortus*) | Ovine/caprine (*B. melitensis*) | Porcine (*B. suis*) | Ovine (*B. ovis*) |
|---|---|---|---|---|
| Albania | - | + | + | + |
| Belgium | + | - | - | - |
| Bulgaria | - | - | + | + |
| Croatia | - | - | + | + |
| Czech Republic | - | - | ? | - |
| France | + | ++ | ? | + |
| Germany | + | - | ? | + |
| Greece | + | ++ | ND | ND |
| Ireland | + | - | - | - |
| Italy | + | + | - | ND |
| Latvia | - | - | + | - |
| Lithuania | - | - | - | ? |
| Macedonia | + | + | - | - |
| Malta | + | + | - | - |
| Poland | + | + | ? | - |
| Portugal | + | + | - | + |
| Romania | - | - | + | - |
| Russia | ++ | ++ | + | + |
| Slovakia | - | - | ND | - |
| Slovenia | - | - | - | + |
| Spain | + | + | - | + |
| Ukraine | ND | ND | ND | ND |
| Yugoslavia | + | + | + | - |

- not present
+ low sporadic incidence
++ high incidence
? presence uncertain
ND no data

None of the four types of brucellosis is present in Austria, Denmark, Estonia, Finland, Hungary, Iceland, Luxembourg, Moldavia, Netherlands, Sweden, Switzerland, and the United Kingdom

Source for Tables 1-8: FAO-WHO-OIE Animal Health Yearbooks, 1994, 1995.

Table 2. Brucellosis in animals, Africa, 1994

| Country | Bovine (*B. abortus*) | Ovine/caprine (*B. melitensis*) | Porcine (*B. suis*) | Ovine (*B. ovis*) |
|---|---|---|---|---|
| Algeria | + | ? | ND | + |
| Angola | ? | ? | ? | ? |
| Botswana | + | ND | - | ND |
| Cape Verde | ? | ? | ? | + |
| Central African Republic | ++ | ND | + | ND |
| Chad | ++ | ? | ? | ND |
| Congo | + | - | - | - |
| Côte d'Ivoire | + | - | - | + |
| Egypt | + | + | ND | - |
| Eritrea | + | ? | ND | + |
| Ghana | + | - | - | - |
| Guinea | + | ND | - | ND |
| Kenya | + | + | ND | ND |
| Libya | + | + | - | - |
| Mauritius | - | - | - | - |
| Morocco | + | ? | - | - |
| Mozambique | ++ | + | ++ | + |
| Namibia | + | - | - | ? |
| Niger | + | + | ND | + |
| Nigeria | ++ | + | + | ND |
| Seychelles | + | - | - | - |
| South Africa | ++ | + | - | + |
| Sudan | ++ | + | - | - |
| Tanzania | + | ND | ND | ND |
| Tunisia | + | ++ | - | - |
| Zaire | + | ND | + | ND |
| Zimbabwe | + | + | - | + |

- not present
+ low sporadic incidence
++ high incidence
? presence uncertain
ND no data

No data on any of the four types of brucellosis are available for Gambia, Mali, and Mauritania

The true incidence of human brucellosis is unknown. Reported incidence in endemic-disease areas varies widely, from <0.01 to >200 per 100,000 population (3). While some areas, such as Peru, Kuwait, and parts of Saudi Arabia, have a very high incidence of acute infections, the low incidence reported in other known brucellosis-endemic areas may reflect low levels of surveillance and reporting, although other factors such as methods of food preparation, heat treatment of dairy products, and direct contact with animals also influence risk to the population.

Consumption of contaminated foods and occupational contact remain the major sources of infection. Examples of human-to-human transmission by tissue transplantation or sexual contact are occasionally reported but are insignificant (4). Prevention of human brucellosis depends on the control of the disease in animals. The greatest success has been achieved in eradicating the bovine disease, mainly in industrialized countries (Table 6); however, most countries have control programs. *B. melitensis* infection has proved more intractable, and success has been limited (Table 7).

Although few recent outbreaks of disease caused by *B. suis* biovar 4 have been reported (5), foci of the infection persist in the Arctic regions of North America and Russia and constitute a potential hazard for the local population. *B. ovis* has not been demonstrated to cause overt disease in humans, although it is widespread in sheep (Tables 1-5). *B. canis* can cause disease in humans, although this is rare even in countries where the infection is common in dogs (6). Precise

Table 3. Brucellosis in animals, Asia, 1994

| Country | Bovine (*B. abortus*) | Ovine/ caprine (*B. melitensis*) | Porcine (*B. suis*) | Ovine (*B. ovis*) |
|---|---|---|---|---|
| Afghanistan | + | + | ND | ND |
| Bangladesh | + | + | ND | ND |
| Bhutan | + | - | - | ND |
| China | + | + | + | + |
| Hong Kong | ND | ND | ? | ND |
| India | + | + | ?+ | - |
| Indonesia | + | ND | + | + |
| Iran | + | + | - | - |
| Israel | - | + | - | - |
| Iraq | + | + | ND | ND |
| Jordan | - | ++ | - | - |
| Korea (S) | ++ | - | ?+ | - |
| Kuwait | ++ | ++ | - | - |
| Malaysia | + | - | ?- | - |
| Mongolia | ++ | + | - | + |
| Myanmar | + | ND | + | ND |
| Oman | ++ | ND | ND | ND |
| Qatar | ND | ND | ND | ND |
| Sri Lanka | ++ | + | - | + |
| Syria | + | ND | ND | ND |
| Thailand | + | - | + | - |
| Turkey | ++ | ++ | - | ND |
| UAE | - | + | - | + |
| Yemen | + | + | - | - |

- not present
+ low sporadic incidence
++ high incidence
? presence uncertain
ND no data
None of the four types of brucellosis is present in Bahrain, Cyprus, Japan, Malaysia (Sabah), Philippines, or Singapore
No data for countries of the former Soviet Union or Qatar

Table 4. Brucellosis in animals, the Americas, 1994

| Country | Bovine (*B. abortus*) | Ovine/ caprine (*B. melitensis*) | Porcine (*B. suis*) | Ovine (*B. ovis*) |
|---|---|---|---|---|
| Antigua/ Barbuda | ? | - | - | - |
| Argentina | ++ | - | + | ++ |
| Belize | - | - | - | ND |
| Bolivia | ++ | + | + | ND |
| Brazil | ++ | - | + | - |
| Canada | - | - | - | + |
| Chile | ++ | - | - | + |
| Colombia | + | - | - | - |
| Cuba | ? | - | ++ | - |
| Dominican Republic | ++ | - | + | - |
| Ecuador | ++ | ND | ND | ND |
| El Salvador | ++ | ND | + | ND |
| Guatemala | + | - | + | - |
| Haiti | + | - | - | - |
| Honduras | ? | - | ++ | - |
| Jamaica | ?+ | - | - | - |
| Mexico | + | + | ND | - |
| Nicaragua | ++ | ND | ND | ND |
| Peru | ++ | ND | ND | ++ |
| Paraguay | + | ND | - | + |
| Uruguay | + | - | - | + |
| United States | + | - | (+) | + |
| Venezuela | ++ | - | ++ | ? |

- not present
+ low sporadic incidence
++ high incidence
? presence uncertain
ND no data
None of the four types of brucellosis is present in Barbados, Falkland Islands, Surinam, or St. Kitts/Nevis information on prevalence is lacking, but *B. canis* has been recorded in the United States, Mexico, Argentina, Spain, China, Japan, Tunisia, and other countries. The recent isolation of distinctive *Brucella* strains, tentatively named *Brucella maris*, from marine animals in the United Kingdom and the United States extends the ecologic range of the genus and, potentially, its scope as a zoonosis (7,8). A hitherto unreported incident of laboratory-acquired infection suggests that this type is pathogenic for humans. Infection could result from occupational contact with infected seals or cetaceans.

### Molecular Genetics

Characterization of the molecular genetics of *Brucella* has taken place almost entirely within the past 10 years. The average molecular complexity of the genome is 2.37 x $10^9$ daltons and the molar G + C 58-59% (9). The genus itself is highly homogeneous with all members showing >95% homology in DNA-DNA pairing studies, thus classifying *Brucella* as a monospecific genus (10). However, the nomenclature proposed by Verger

Table 5. Brucellosis in animals, Oceania, 1994

| Country | Bovine (*B. abortus*) | Ovine/ caprine (*B. melitensis*) | Porcine (*B. suis*) | Ovine (*B. ovis*) |
|---|---|---|---|---|
| Australia | - | - | (+) | + |
| Cook Island | - | ND | - | ND |
| New Caledonia | - | - | - | - |
| New Zealand | - | - | - | ++ |
| Samoa | + | ND | ND | ND |

++ high prevalence
+ present
(+) limited presence
- not present
ND no data
None of the four types of brucellosis is present in Vanuatu

Table 6. Countries reporting eradication of bovine brucellosis, 1994

| | | |
|---|---|---|
| EUROPE | | |
| Bulgaria (1958) | Croatia (1965) | Czech Republic (1964) |
| Denmark (1962) | Estonia (1961) | Finland (1960) |
| Hungary (1985) | Iceland (never recorded) | Latvia (1963) |
| Lithuania (1952) | Luxembourg (1993) | Netherlands (1993) |
| Romania (1969) | Slovak Republic (1964) | Slovenia (1970) |
| Sweden (1957) | Switzerland (1963) | U.K. (1993) |
| AFRICA | | |
| Mauritius (1986) | | |
| AMERICAS | | |
| Belize (1980) | Canada (1989) | |
| ASIA | | |
| Cyprus (1932) | Israel (1984) | Japan (1992) |
| Jordan (1992) | N Korea (1959) | Papua New Guinea (1974) |
| Philippines (1989) | U.A.E. (1992) | |
| OCEANIA | | |
| Australia (1989) | French Polynesia (1984) | |
| New Zealand (1989) | Vanuatu (1992) | |

and colleagues, in which all types would be regarded as biovars of *B. melitensis*, has not been generally adopted on practical grounds. For this reason, although its shortcomings are well known, the old nomenclature has been retained with the former species' names *B. abortus*, *B. melitensis*, *B. suis*, *Brucella neotomae*, *B. ovis*, and *B. canis* being used for the corresponding nomen species (11,12). Within these, seven biovars are recognized for *B. abortus* (1,7-10,12,13), three for *B. melitensis* (1, 7,8), and five for *B. suis* (1,7-10,12). The other species have not been differentiated into biovars, although variants exist (14). The current biotyping system does not encompass all known variants even of the principal species. Thus, variants of *B. melitensis* have been described; this suggests that the scheme should be extended (11,13,15). The strains isolated from marine animals clearly form a separate group and have been unofficially designated *B. maris* (E. S. Broughton, unpub. data). At least two subdivisions of this strain can be distinguished, corresponding approximately to strains isolated from cetaceans and seals, respectively (7,8).

Restriction fragment patterns produced by infrequently cutting endonucleases provide support for the current differentiation of the nomen species (16). Restriction endonuclease analysis has generally been unsuccessful for typing when applied to the whole genome (17) but polymerase chain amplification of selected sequences followed by restriction analysis has provided evidence of polymorphism in a number of genes including *omp 2*, *dnaK*, *htr*, and *ery* (the erythrulose-1-phosphate dehydrogenase gene) (18-20). The *omp2* gene is taxonomically important because it determines dye sensitivity, one of the traditional typing methods for biovar differentiation (21). Its polymorphism and capacity for posttranslational modification of its product may explain the tendency for variation in dye sensitivity patterns and have been used as the basis for a genetic classification of *Brucella* (22,23). The *dnaK* gene

Table 7. Countries reporting eradication of other forms of brucellosis, 1994

| Region | Ovine/caprine (*B. melitensis*) | Porcine (*B. suis*) | Ovine (*B. ovis*) |
|---|---|---|---|
| Europe | | | |
| | Bulgaria (1941) | Denmark (1951) | Czech Rep. (1951) |
| | Croatia (1991) | Estonia (1988) | Germany (1986) |
| | Czech Rep. (1951) | Lithuania (1991) | Latvia (1989) |
| | Germany (1986) | Sweden (1957) | |
| | Switzerland (1963) | | |
| Africa | | | |
| | Ghana (1993) | None | Ghana (1993) |
| | Namibia (1990) | | |
| Americas | | | |
| | United States (1972) | Belize (1985) | Falkland Is. (1991) |
| | Chile (1987) | Honduras (1992) | |
| | | Colombia (1982) | Mexico (1991) |
| Asia | | | |
| | Cyprus (1993) | Singapore (1989) | Yemen (1989) |
| Oceania | | | |
| | Not present | None | None |

of *B. melitensis* is cleaved into two fragments by Eco RV endonuclease, whereas the genes of the other nomen species all produce a single fragment (24). The *ery* gene is reported to have undergone a 7.2 kbp deletion in *B. abortus* strain 19 (20). This could explain this strain's erythritol sensitivity, a major factor in its attenuation.

The genome of *Brucella* contains two chromosomes of 2.1 and 1.5 mbp, respectively. Both replicons encode essential metabolic and replicative functions and hence are chromosomes and not plasmids (25,26). Natural plasmids have not been detected in *Brucella*, although transformation has been effected by wide host range plasmids after conjugative transfer or electroporation (27).

rRNA sequencing has defined the phylogenetic relationship of *Brucella*. Its closest known relation, *Ochrobactrum anthropi*, is an environmental bacterium associated with opportunistic infections (28); this organism is also detected by a polymerase chain reaction (PCR) procedure that is otherwise specific for *Brucella* (29). Possibly more closely related is the incompletely characterized *Vibrio cyclosites*, which displays >90% similarity of 5S rRNA sequence (30). Less closely related but within the same subgroup of the -2 Proteobacteria are *Agrobacterium*, *Phyllobacterium*, and *Rhizobium*, which also possess multiple replicons and a capacity for intracellular growth. The *Bartonella* group also shows some affinity to *Brucella* on the basis of rRNA, but not DNA, similarity (31). Other similarities have been noted in cell membrane lipid composition and intracellular growth.

### Antigenic Composition

A substantial number of antigenic components of *Brucella* have been characterized. However, the antigen that dominates the antibody response is the lipopolysaccharide (LPS). In smooth phase strains (S), the S-LPS comprises a lipid A (containing two types of aminoglycose); distinctive fatty acids (excluding ß-hydroxymyristic acid); a core region containing glucose, mannose, and quinovosamine; and an O chain comprising a homopolymer of approximately 100 residues of 4-formamido-4,6-dideoxymannose (linked predominantly α-1,2 in A epitope-dominant strains with every fifth residue linked α-1,3 in M dominant strains) (32).

The difference in linkage influences the shape of the LPS epitopes. The A-dominant type is rod-shaped and is determined by five consecutive α-1,2 linked residues, whereas the M-dominant type is kinked and determined by four residues, including one linked α-1,3 (33). Strains that react with antisera to both A and M epitopes produce LPS of both types in approximately equal proportions (30), consistent with the origi-nal hypothesis of Wilson and Miles (34). The presence of 4-amino, 4,6 dideoxymannose in the LPS is also responsible for the antigenic cross-reactivity with *Escherichia hermanni* and *Escherichia coli* O:157, *Salmonella* O:30, *Stenotrophomonas maltophilia*, *Vibrio cholerae* O:1, and *Yersinia enterocolitica* O:9 LPS (32). The structure of the LPS of nonsmooth strains (R-LPS) is basically similar to that of the S-LPS except that the O-chain is either absent or reduced to a few residues. The specificity of the R-LPS is, therefore, largely determined by the core polysaccharide.

Numerous outer and inner membrane, cytoplasmic, and periplasmic protein antigens have also been characterized. Some are reognized by the immune system during infection and are potentially useful in diagnostic tests (35). Hitherto, tests based on such antigens have suffered from low sensitivity as infected persons tend to develop a much less consistent response to individual protein antigens than to LPS. Thus, tests such as immunoblotting against whole-cell extracts may have some advantages over more quantitative tests that employ purified individual antigens (36).

Recently, ribosomal proteins have reemerged as immunologically important components. Interest in these first arose more than 20 years ago when crude ribosomal preparations were demonstrated to stimulate both antibody and cell-mediated responses and to confer protection against challenge with *Brucella* (37). However, the individual components responsible for such activity were not identified until recently. It has been established that the L7/L12 ribosomal proteins are important in stimulating cell-mediated responses. They elicit delayed hypersensivity responses as components of brucellins (38), and as fusion proteins, they have been shown to stimulate protective responses to *Brucella* (39). They appear to have potential as candidate vaccine components.

### Mechanisms of Pathogenicity

Virulent *Brucella* organisms can infect both nonphagocytic and phagocytic cells. The mechanism of invasion of nonphagocytic cells is not clearly established. Cell components specifically promoting cell adhesion and invasion have not been characterized, and attempts to detect invasin genes homologous to those of enterobacteria have failed. Within nonphagocytic cells, brucellae tend to localize in the rough endoplasmic reticulum. In polymorphonuclear or mononuclear phagocytic cells, they use a number of mechanisms for avoiding or suppressing bactericidal responses. The S-LPS probably plays a substantial role in intracellular survival, as smooth organisms survive much more effectively than nonsmooth ones. Compared with enterobacterial LPS, S-LPS has many unusual properties: a relatively low toxicity for endotoxin-sensitive mice, rabbits, and chick embryos; low toxicity for macrophages; low pyrogenicity; and low hypoferremia-inducing activity. It is also a relatively poor inducer of interferon (and tumor necrosis factor) but, paradoxically, is an effective inducer of interleukin 12 (40,41).

S-LPS is the main antigen responsible for containing protection against infection in passive transfer experiments with monoclonal and polyclonal antibodies. The protection is usually short-term and incomplete, however. The elimination of virulent *Brucella* depends on activated macrophages and hence requires development of Th1 type cell-mediated responses to protein antigens (42).

An important determinant of virulence is the production of adenine and guanine monophosphate, which inhibit phagolysosome fusion; degranulation and activation of the myeloperoxidase-halide system; and production of tumor necrosis factor (41,43). The production of these inhibitors is prevented in *pur E* mutants, which are substantially attenuated in consequence. Cu-Zn superoxide dismutase is believed to play a significant role in the early phase of intracellular infection (44). However, conflicting results have been reported, and this role needs to be confirmed.

Survival within macrophages is associated with the synthesis of proteins of molecular weight 17, 24, 28, 60, and 62 kDa. The 62 kDa protein corresponds to the Gro EL homologue Hsp 62, and the 60 kDa protein is an acid-induced variant of this. The 24 kDa protein is also acid-induced, and its production correlates with bacterial survival under acidic conditions (<pH4). The 17 and 28 kDa proteins are apparently specifically induced by macrophages and correlated with intracellular survival (45).

Another stress-induced protein, HtrA, is involved in the induction of an early granulomatous response to *B. abortus* in mice and is associated with a reduction in the levels of infection during the early phase. Howevr, it does not prevent a subsequent increase in bacterial numbers, and *htrA*-deficient mutants ultimately produce levels of splenic infection similar to those given by wild-type *B. abortus* (46). Similarly, *recA*-deleted mutants produce a lower initial spleen count than *recA*-positive strains but still establish persistent infection (47). The role of iron-sequestering proteins or other siderophores in the pathogenesis of brucellosis is still unknown. In general, the low availability of iron in vivo restricts microbial growth. However, high iron concentrations promote the killing of *Brucella*, probably by favoring production of hydroxylamine and hydroxyl radical.

The mechanisms of pathogenesis of *Brucella* infection in its natural host species and in humans are still not completely understood, and further studies are needed.

### Diagnosis

The clinical picture in human brucellosis can be misleading, and cases in which gastrointestinal, respiratory, dermal, or neurologic manifestations predominate are not uncommon (48-52). Because unusual cases with atypical lesions continue to be reported, diagnosis needs to be supported by laboratory tests (52). Blood culture is still the standard method and is often effective during the acute phase; the lysis concentration method gives the best results (53). Automated incubation-detection methods are effective, but allowance should be made for the relatively slow growth of the organism (54). Presumptive identification is made on the basis of morphologic, cultural, and serologic properties. Confirmation requires phage-typing, oxidative metabolism, or genotyping procedures. Reliance should not be placed on gallery type rapid identification systems as these have misidentified *Brucella* as *Moraxella phenylpyruvica*, with serious consequences for laboratory staff (55).

PCR with random or selected primers gives promising results, but standardization and further evaluation are needed, especially for chronic disease (56). Similarly, antigen detection methods are potentially useful but have not been validated. Combinations of these with PCR, such as immuno-PCR, have considerable potential but require evaluation. Enzyme immunoassay is now widely used for serologic diagnosis of the disease in humans and other species. IgA and IgG antibodies seem the most useful indicators of active infection (57,58). Western blotting against selected cytoplasmic proteins may be useful in support of screening tests to differentiate active from past or subclinical infection (35).

### Treatment

Despite extensive studies over the past 15 years, the optimum antibiotic therapy for brucellosis is still disputed. The treatment recommended by the World Health Organization for acute brucellosis in adults is rifampicin 600 to 900 mg and doxycycline 200 mg daily for a minimum of 6 weeks (59). Some still claim that the long-established combination of intramuscular streptomycin with an oral tetracycline gives fewer relapses (60). There is some evidence of physiologic antagonism between rifampicin and tetracyclines, but recent studies suggest that the two regimens have very similar results given adequate time. Quinolones in combination with rifampicin seem as effective as either of these regimens (61). Controlled clinical trials with other antibiotics, including new macrolides and ß-lactams, have either give inferior results or involved too few patients for proper evaluation.

Infections with complications, such as meningoencephalitis or endocarditis, require combination therapy with rifampicin, a tetracycline, and an aminoglycoside (62). Rifampicin has been recommended as the treatment of choice for uncomplicated disease in children, with cotrimoxazole as an alternative. Both are associated with a high relapse rate if used singly, and best results are achieved by using them in combination (63). Cotrimoxazole is an alternative but also has a high relapse rate. A combination of the two agents gives the best results.

### Prevention

Prevention of brucellosis in humans still depends on the eradication or control of the disease in animal hosts, the exercise of hygienic precautions to limit exposure to infection through occupational activities, and the effective heating of dairy products and other potentially contaminated foods. Vaccination now has only a small role in the prevention of human disease, although in the past, various preparations have been used, including the live attenuated *B. abortus* strains 19-BA and 104M (used mainly in the former Soviet Union and China), the phenol-insoluble peptidoglycan vaccine (formerly available in France), and the polysaccharide-protein vaccine (used in Russia). All had limited efficacy (64) and in the cases of live vaccines, were associated with potentially serious reactogenicity. Subunit vaccines against brucellosis are still of interest. The live vaccines have provoked unacceptable reactions in individuals sensitized by previous exposure to *Brucella* or if inadvertently administered by subcutaneous rather than percutaneous injection. These will probably require a combination of detoxified lipopolysaccharide-protein conjugate and protein antigens such as the L7/L12 ribosomal proteins presented in an adjuvant or delivery system favoring a Th1 type immune response. *pur E* mutants of *B. melitensis* appear safe in animals (65) and may have potential application as human vaccines if their safety and efficacy is confirmed in clinical trials. New vaccines have been evaluated for use in animals, including the *B. suis* strain 2 live vaccine given either orally or parenterally (66,67). This vaccine has proved inferior to the Rev.1. strain for the prevention of *B. melitensis* infection in sheep and goats and ineffective against *B. ovis* infection in sheep. *B. abortus* strain 19 still appears to be as effective as any for the prevention of *B. abortus* infection in cattle. However, the RB51 strain of *B. abortus*, an R mutant used as a live vaccine, has been licensed in the United States. This does not interfere with diagnostic serologic tests, but in laboratory trials, its efficacy appeared comparable with that of strain 19 (68). Similar *rfb* mutants of *B. melitensis* and *B. suis* are under development for the prevention of ovine/caprine and porcine brucellosis.

Substantial progress has been achieved in understanding the molecular basis of the genetics of *Brucella* and the pathogenesis of the infection. However, further progress is needed, especially in relation to diagnostic procedures and therapy. An effective and safe vaccine against human brucellosis is also some way in the future.

**Michael J. Corbel**
National Institute of Biological Standards
and Control, Hertfordshire, United Kingdom

### References

1. Amato Gauci AJ. The return of brucellosis. Maltese Medical Journal 1995;7:7-8.

2. Garcia Carrillo C. Animal and human brucellosis in the Americas. Paris: OIE, 1990: 287.
3. Lopez Merino A. Brucellosis in Latin America. Young EJ, Corbel MH, editors. Brucellosis; clinical and laboratory aspects. Boca Raton: CRC Press Inc., 1989:151-61.
4. Mantur BG, Mangalgi SS, Mulimani B. *Brucella melitensis*-a sexually transmissible agent. Lancet 1996;347:1763.
5. Tessaro SV, Forbes LB. *Brucella suis* biotype 4; a case of granulomatous nephritis in a barren ground caribou (*Rangifer tarandus groenlandicus L*) with a review of the distribution of rangiferine brucellosis in Canada. J Wildlife Dis 1986;22:479-88.
6. Carmichael LE. *Brucella canis*. In: Nielsen K, Duncan JR, editors. Animal brucellosis. Boca Raton: CRC Press Inc.: 1990,335-50.
7. Ross HM, Foster G, Reid RJ, Jabans KL, MacMillan AP. *Brucella* infection in sea mammals. Vet Rec 1994;132:359.
8. Ewalt DR, Payeur JB, Martin BM, Cummins DR, Miller WG. Characteristics of a *Brucella* species from a bottlenose dolphin (*Tursiops truncatus*). J Vet Diagn Invest 1994;6:448-52.
9. De Ley J, Mannheim W, Segers P, Lievens A. Ribosomal ribonucleic acid cistron similarities and taxonomic neighbourhood of *Brucella* and CDC Group Vd. Int J Syst Bacteriol 1987;37:35-42.
10. Verger JM, Grimont F, Grimont PAD, Grayon M. *Brucella* A monospecific genus as shown by deoxyribonucleic acid hybridization. Int J Syst Bacteriol 1985;35:292-5.
11. Alton GG, Jones LM, Pietz DE. Laboratory techniques in brucellosis. Geneva: World Health Organization 1975.
12. Corbel MJ, Morgan WJB. Genus *Brucella* Meyer and Shaw 1920, 173 AL. In: Holt JG, editor. Bergey's manual of systematic bacteriology vol. 1. Baltimore (MD): Williams and Wilkins, 1984:377-88.
13. Corbel MJ. Identification of dye-sensitive strains of *Brucella melitensis.* J Clin Microbiol 1991;29:1066-8.
14. Corbel MJ, Thomas EL. Use of phage for the identification of *Brucella canis* and *Brucella ovis* cultures. Res Vet Sci 1985;35:35-40.
15. Banai M, Mayer I, Cohen A. Isolation, identification and characterization in Israel of *Brucella melitensis* biovar 1 atypical strains susceptible to dyes and penicillin, indication of the evolution of a new variant. J Clin Microbiol 1990;28:1057-9.
16. Allardet-Servent A, Bourg G, Ramuz M, Bellis M, Roizes G. DNA polymorphism in strains of the genus *Brucella.* J Bacteriol 1988;170:4603-7.
17. O'Hara MJ, Collins DM, Lisle GW. Restriction endonuclease analysis of *Brucella ovis* and other *Brucella* species. Vet Microbiol 1985;10:425-9.
18. Ficht TA, Bearden SW, Sowa BA, Adams LG. DNA sequence and expression of the 36-kilodalton outer membrane protein gene of *Brucella abortus.* Infect Immun 1989;57:3281-91.
19. Cellier MFM, Teyssier J, Nicolas M., Liautard JB, Marti J, SriWidada J. Cloning and characterization of the *Brucella ovis* heat shock protein DnaK functionally expressed in *Escherichia coli.* J Bacteriol 1992;174:8036-42.
20. Sangari FJ, García-Lobo JM, Aguero J. The *Brucella abortus* vaccine strain B19 carries a deletion in the erythritol catabolic genes. FEMS Microbiol Lett 1994;121:337-42.
21. Douglas JT, Rosenberg EY, Nikaido H, Verstreate DR, Winter AJ. Porins of *Brucella* species. Infect Immun 1984;44:16-21.
22. Ficht TA, Husseinen HS, Derr J, Bearden SW. Species-specific sequences at the omp2 locus of *Brucella* type strains. Int J Syst. Bacteriol 1996;46:329-31.
23. Cloeckaert A, Verger J-M, Grayon M, Grepinet O. Restriction site polymorphism of the genes encoding the major 25kDa and 36kDa outer membrane proteins of *Brucella.* Microbiology 1995;141:2111-21.
24. Cloeckaert A, Salih-Alj Debarrh H, Zygmunt MS, Dubray G. Polymorphism at the *dnak* locus of *Brucella* species and identification of a *Brucella melitensis* species-specific marker. J Med Microbiol 1996;45:200-13.
25. Michaux S, Paillisson J, Carles-Nurit MJ, Bourg G, Allardet Servent A, Ramuz M. Presence of two independent chromosomes in the *Brucella melitensis* 16M genome. J Bacteriol 1993;175:701-5.
26. Jumas-Bitlak E, Maugard C, Michaux-Charachon S, Allardet-Servent A, Perrin A, O'Callaghan D. Study of the organization of the genomes of *Escherichia coli, Brucella melitensis* and *Agrobacterium tumefaciens* by insertion of a unique restriction site. Microbiology 1995;141:2425-32.
27. Rigby CE, Fraser ADE. Plasmid transfer and plasmid-mediated genetic exchange in *Brucella abortus.* Can J Vet Res 1989;53:326-30.
28. Cieslak TJ, Robb ML, Drabick CJ, Fisher GW. Catheter-associated sepsis caused by *Ochrobactrum anthropi*: report of a case and review of related non-fermentative bacteria. Clin Infect Dis 1992;14:902-7.
29. Da Costa M, Guillou J-P, Garin-Bastuji B, ThiJbaud M, Dubray G. Specificity of six gene sequences for the detection of the genus *Brucella* by DNA amplification. J Appl Bacteriol 1996;81:267-75.
30. Minnick M F, Stiegler G L. Nucleotide sequence and comparison of the 5S ribosomal genes of *Rochalimaea henselae, R. quintana* and *Brucella abortus.* Nucleic Acids Res 1993;21:2518.
31. Relman DA, Lepp PW, Sadler KN, Schmidt TM. Phylogenetic relationships among the agent of bacillary angiomatosis, *Bartonella bacilliformis*, and other alpha-proteobacteria. Mol. Microbiol 1992;6:1801-7.
32. Perry MB, Bundle DR. Lipopolysaccharide antigens and carbohydrates of *Brucella.* In: Adams LG, editor. Advances in Brucellosis Research Austin (TX): Texas A & M University, 1990;76-88.
33. Bundle DR, Cherwonogrodzky JW, Gidney MAJ, Meikle PJ, Perry MB, Peters T. Definition of *Brucella* A and M epitopes by monoclonal typing reagents and synthetic oligosaccharides. Infect Immun 1989;57:2829-36.
34. Wilson GS, Miles AA. The serological differentiation of smooth strains of the *Brucella* group. British Journal of Experimental Pathology 1932;13:1-13.

35. Goldbaum FA, Leoni J, Walach JC, Fossati CA. Characterisation of an 18-kilodalton *Brucella* cytoplasmic protein which appears to be a serological marker of active infection of both human and bovine brucellosis. J Clin Microbiol 1993;31:2141-5.
36. Goldbaum FA, Morelli L, Wallach J, Rubbi CP, Fossati CA. Human brucellosis: immunoblotting analysis of three *Brucella abortus* antigenic fractions allows the detection of components of diagnostic importance. Medicina 1991;51:227-32.
37. Corbel MJ. The immunogenic activity of ribosomal fractions derived from *Brucella abortus*. Journal of Hygiene, Cambridge 1976;76:65-74.
38. Bachrach G, Banai M, Bardenstein S, Hoida G, Genizi A, Bercovier H. Brucella ribosomal protein L7 / L12 is a major component in the antigenicity of Brucellin INRA for delayed hypersensitivity in *Brucella*-sensitized guinea- pigs**.** Infect Immun 1994;62:5361-6.
39. Oliveira S, Splitter GA. Immunization of mice with recombinant L7 / L12 ribosomal protein confers protection against *Brucella abortus* infection. Vaccine 1996;14:959-62.
40. Zhan Y, Cheers C**.** Differential activation of *Brucella*-reactive CD4+ cells by *Brucella* infection or immuni-zation with antigenic extracts. Infect Immun 1995;63:969-95.
41. Caron E, Peyrard T, Kohler S, Cabane S, Liautard J-P, Dornand J. Live *Brucella* spp. fail to induce tumour necrosis factor alpha excretion upon infection of U937-derived phagocytes. Infect Immun 1994;62:5267-74.
42. Dubray G. Protective antigens in brucellosis. Annales de l'Institut Pasteur, Microbiologie 1987;138:84-7.
43. Canning PC, Roth JA, Deyoe BL. Release of 5'-guanosine monophosphate and adenine by *Brucella abortus* and the intracellular survival of the bacteria. J Infect Dis 1986; 154:464-70.
44. Bricker BJ, Tabatabai LB, Judge BA, Deyoe BL, Mayfield JE. Cloning, expression and occurrence of the *Brucella* Cu-Zn dismutase. Infect Immun 1990;58:2933-9.
45. Lin J, Ficht TA. Protein synthesis in *Brucella abortus* induced during macrophage infection. Infect Immun 1995;63:1409-14.
46. Tatum FM, Cheville NF, Morfitt D. Cloning, characterisation and construction of *htr A* and htr A - like mutants of *Brucella abortus* and their survival in BALB/C mice. Microb Pathog 1994;17:23-36.
47. Tatum FM, Morfitt DC, Halling SM. Construction of a *Brucella abortus* Rec A mutant and its survival in mice. Microb Pathog 1993;14:177-85.
48. Santini C, Baiocchi P, Berardelli A, Venditti M, Serra P. A case of brain abscess due to *Brucella melitensis.* Clin Infect Dis 1994;19:977-8.
49. Potasman I, Even L, Banai M, Cohen E, Angel D, Jaffe M. Brucellosis: an unusual diagnosis for a seronegative patient with abscesses, osteomyelitis and ulcerative colitis. Rev Clin Dis 1991;13:1039-42.
50. Shakir RA, Al-Din ASN, Araj GF, Lulu AR, Mousa AR, Saadah MA. Clinical diagnosis of neurobrucellosis. A report on 19 cases. Brain 1987;110:213-23.
51. Young EJ. An overview of human brucellosis. Clin Infect Dis 1995;21:283-90.
52. Madkour MM. Brucellosis. Butterworths, London 1989.
53. Kolman S, Maayan MC, Gotesman G, Roszenstain LA, Wolach B, Lang R. Comparison of the Bactec and lysis concentration method for the recovery of *Brucella* species from clinical specimens. Eur J Clin Microbiol Infect Dis 1991;10:647-8.
54. Solomon HM, Jackson D. Rapid diagnosis of *Brucella melitensis* in blood; some operational characteristics of the BACT / ALERT. J Clin Microbiol 1992;28:2139-41.
55. Luzzi GA, Brindle R, Socket PN, Solera J, Klenerman P, Warrell DA. Brucellosis: imported and laboratory-acquired cases, and an overview of treatment trials. Trans Roy Soc Trop Med Hyg 1993;87:138-41.
56. Matar FM, Khreissir IA, Abdonoor AM. Rapid laboratory confirmation of human brucellosis by PCR analysis of a target sequence on the 31-kilodalton *Brucella* antigen DNA. J Clin Microbiol 1996;34:477-8.
57. Araj GF, Lulu AR, Mustafa MY, Khateeb MI. Evaluation of ELISA in the diagnosis of acute and chronic brucellosis in human beings. Journal of Hygiene, Cambridge 1986;97:457-69.
58. Ariza J, Pellicer T, Pallarés R, Foz A, Gudiol F. Specific antibody profile in human brucellosis. Clin Infect Dis 1992;14:131-40.
59. Joint FAO / WHO Expert Committee on Brucellosis. Sixth Report. World Health Organ Tech Rep Ser No. 740. Geneva: World Health Organization, 1986.
60. Ariza J, Gudiol F, Pallarés R, Rufi G, Fernàndez-Viladrich P. Comparative trial of rifampicin-doxycycline versus tetracycline-streptomycin in the therapy of human brucellosis. Antimicrob Agents Chemother 1985;28:548-51.
61. Akova M, Uzun O, Akalin HE, Hayran M, Unal S, Gur D. Quinolones in the treatment of human brucellosis; comparative trial of ofloxacin-rifampin versus doxycycline-rifampin. J Antimicrob Chemother 1993;37:1831-4.
62. Shakir RA. Neurobrucellosis. Postgrad Med J 1986;62:1077-9.
63. Khuri-Bulos NA, Daoud AH, Azab SM. Treatment of childhood brucellosis: results of a prospective trail on 113 childred. Ped Infect Dis 1993;12:377-81.
64. Corbel MJ. Vaccines against bacterial zoonoses. J Med Microbiol 1997;46:267-9.
65. Crawford RM, Van De Verg L, Yuan L, Hadfield TL, Warren RL, Drazek ES, et al. Deletion of *purE* attenuates *Brucella melitensis* infection in mice. Infect Immun 1996;64:2188-92.
66. Xie X. Orally administered brucellosis vaccine *Brucella suis* strain 2 vaccine. Vaccine 1986;4:212-6.
67. Mustafa AA, Abusowa M. Field-oriented trial of the Chinese *Brucella suis* strain 2 vaccine in sheep and goats in Libya. Annales de Recherche Veterinaire 1993;24:422-9.
68. Schurig GG, Roop RM, Bagchi T, Boyle S, Buhrman D, Sriranganathan N. Biological properties of RB51. A stable strain of *Brucella abortus*. Vet Microbiol 1991;28:171-88.
